# The effectiveness of robotic-assisted upper limb rehabilitation to improve upper limb function in patients with cervical spinal cord injuries: a systematic literature review

**DOI:** 10.3389/fneur.2023.1126755

**Published:** 2023-08-09

**Authors:** Jocelyn Sze-wing Ho, Koko Shaau-yiu Ko, Sheung Wai Law, Gene Chi-wai Man

**Affiliations:** Department of Orthopaedics and Traumatology, The Faculty of Medicine, The Chinese University of Hong Kong, The Prince of Wales Hospital, Hong Kong SAR, China

**Keywords:** limb activation, robot-assisted therapy, spinal cord injury, exoskeleton, adults

## Abstract

**Background:**

Spinal Cord Injury (SCI) damages corticospinal tracts and descending motor pathways responsible for transmitting signals from the brain to the spinal cord, leading to temporary or permanent changes in sensation, motor function, strength, and body function below the site of injury. Cervical SCI (cSCI), which leads to tetraplegia, causes severe functional upper limb (UL) impairments that increase falls risk, limits independence, and leads to difficulties with activities of daily living (ADLs). Robotic therapy (RT) has been developed in recent decades as a new treatment approach for people with cervical spinal cord injuries (cSCI). The present review aimed to explore current available evidence and studies regarding the effectiveness of RT for individuals with cSCI in improving UL function, identify current research gaps and future research directions.

**Method:**

This review was conducted by searching PubMed, CINAHL, Medline, Embase, and APA PsycInfo for relevant studies published from January 2010 to January 2022. Selected studies were analyzed with a focus on the patients’ self-perception of limited UL function and level of independence in activities of daily living. In addition, the JBI Critical Appraisal checklist was used to assess study quality.

**Results:**

A total of 7 articles involving 87 patients (74 males and 13 females) were included in the analysis, with four studies utilizing exoskeleton and three studies utilizing end-effector robotic devices, respectively. The quality of these studies varied between JBI Critical Appraisal scores of 4 to 8. Several studies lacked blinding and a control group which affected internal validity. Nevertheless, four out of seven studies demonstrated statistically significant improvements in outcome measurements on UL function and strength after RT.

**Conclusion:**

This review provided mixed evidence regarding the effectiveness of RT as a promising intervention approach to improve upper limb function in participants with cSCI. Although RT was shown to be safe, feasible, and reduces active therapist time, further research on the long-term effects of UL RT is still needed. Nevertheless, this review serves as a useful reference for researchers to further develop exoskeletons with practical and plausible applications toward geriatric orthopaedics.

## 1. Introduction

Spinal cord injury (SCI) is a devastating, life-changing event that occurs due to trauma of the vertebral column or its surrounding tissue, which causes damage to corticospinal tracts and descending motor pathways and ascending sensory pathways are responsible for transmitting signals from the brain to the spinal cord (1–3). Often, this can leads to temporary or permanent loss in sensation, motor function, strength, and body function below the site of injury (4). Previous demographics showed the annual incidence rate of SCI in the United States was 54 cases per million population (5), with the prevalence rate of 721 to 906 per million people (6). Among these SCIs, cervical SCI (cSCI) is the most common occurrence. It makes up around 62% of all SCIs to cause severe functional upper limb (UL) impairments and difficulties with activities of daily living (ADLs) (7). This may also lead to tetraplegia. Hence, through the restoration of UL function, including range of movement (ROM) and muscle strength in the arms and hands, patients can regain independence and improve their quality of life (8, 9).

Our arm plays an important role to maintain balance following a postural disturbance (10). Disturbance of arm swing in non-impaired adults during walking have resulted to alter temporal–spatial gait parameters and interrupt natural pelvic-thoracic motion (11). Importantly, bilateral arm swing restriction have been shown to increase the metabolic cost of walking, impairing stability and increasing fall risk through inducing physical fatigue (12, 13). Hence, the arms are important for locomotor stability and preventing falls by controlling whole-body angular momentum, redirecting the body’s center-of-mass, and providing support to arrest descent.

Recent approaches in allied-health interventions have demonstrated modest evidence to preserve the range of motion and enhance mobility skills in the UL. Currently, there are more than 120 devices being developed for UL rehabilitation toward patients affected by neurologic disability (14). These rehabilitation regimes comprise of repetitive movement patterns, functional exercises, verbal and visual feedback, and task-oriented training are considered effective in improving upper limb function (15). However, these newer interventions, such as robotic-assisted upper limb rehabilitation, remains to be accounted as experimental (16). This is owing to the variation of the training characteristics, the type of training and the absence of specific outcome measures to limit the applicability of evidence. In addition, the optimization of robotic-assisted upper limb rehabilitation for maximizing functional improvements (i.e., ADLs, quality of life, activities, and participation) and preserving and/or increasing such progress over time is still an open question. Moreover, the characterization of the type of patient that could benefit from the treatment with different robotic systems remains poorly explored. Although Lu et al. outlined current rehabilitation options to improve UL function in patients with spinal cord injuries, further research are still needed to determine the effectiveness of robotic rehabilitation (17).

Robotic assisted UL rehabilitation, or robotic therapy (RT), facilitates UL function by assisting in repetitive labor-intensive manual therapy normally administered by a physiotherapist (PT) or occupational therapist (OT) (18). Such that, UL robotic devices increase the number of motor repetitions to aid patient recovery and provide consistent training to measure performances outcomes (19). Unlike traditional hands-on therapy, RT would not lack frequency and intensity due to labor limitations and cost (20). Additionally, traditional hands-on rehabilitation outcomes may differ based on the variation in practice between therapists. Robotic devices are either categorized as end-effector-based or exoskeletons. End-effector-based devices are adaptable to patients of various sizes, and exoskeleton-based devices require specific modifications due to optimal joint adaptations (21). While for exoskeletons, they can be classified into grounded exoskeletons and wearable exoskeletons (22). These design approaches affect the level of control over the interaction as well as the output impedance of the device and the ability to modulate this impedance through control. These requires large reduction ratios and results in high inertia and friction at the output where the patient is attached, which can partially be compensated through control. Many researchers have investigated UL rehabilitation according to the facilitation approach with increased physical therapy, electrical stimulation, and passive manipulation (23–25). Toward the clinical evaluations, these include scales for the upper limb function (e.g., using the Fugl-Meyer and the Motricity Index), spasticity, and health-related quality of life questionnaires toward daily activities (26, 27). And the evaluation of muscle strength and the finger pinch are common instrumental assessments (28, 29). Despite various robotic assisted therapy devices being developed since the 1990s, there are still no standardized protocols around the use of these devices in patients with spinal cord injuries. Additionally, while systematic reviews focusing on robotic lower limb rehabilitation were widely published, reviews appraising relevant evidence around the effectiveness of upper limb robotic rehabilitation for individuals with cSCIs are still lacking.

Even though there was a published systematic review around the use of UL robotic devices, the inclusion of low quality appraised studies affected the overall quality of the review (30). Though Morone et al. also have published a comprehensive review toward the state-of-the-art clinical applications around UL robotic training in motor and functional recovery for cSCI patients (31), the search strategy was not explicit due to the limited inclusion and exclusion criteria. Herein, the present review aimed to explore current available evidence and studies regarding the effectiveness of robotic-assisted therapy for individuals with cSCI in improving UL function, and to identify current research gaps for future research directions.

## 2. Methods and methods

The current systematic review was conducted according to the recommendations in the Preferred Reporting Items for Systematic Reviews and Meta-analysis (PRISMA) Statement (32). No ethical approval was needed because all analyses were based on published evidence.

### 2.1. Data sources and search strategy

The systematic literature review was performed by searching the following database: PubMed, Cumulative Index of Nursing and Allied Health Literature (CINAHL), Medline, Embase, and APA PsycInfo. To understand the changes for the past decade, we included relevant articles published from January 2010 to January 2022. The following keywords were used for the literature search: (“Robotic therapy or Robotic assisted training or robotic assisted therapy or robot* or exoskeleton or telerobot* or wrist-robot* or robotic upper limb rehabilitation”) AND (“adult* or patient or individual* or young person* or young adult* or person or elderly or aged or older or elder* or geriatric* or elderly people or old people or older people or senior*”) AND (“cervical sci or cervical spine cord* or central cord syndrome or central spinal cord or central spinal cord injur* or ccs”) AND (upper limb or upper limb function or arm function or hand function or upper extremit* or upper extremity function). The full search strategy and key search terms can be found in Supplementary 1. Subsequently, the reference lists were manually screened by two independent expert observers to reach a common agreement on relevant studies.

### 2.2. Study eligibility criteria

Studies were included when the following criteria were met: (1) articles that report findings regarding the effectiveness of robotic-assisted therapy application in human subjects; (2) allied health prescribed robotic therapy to aid upper limb training; (3) quantitative investigation on the outcome of improving upper limb function; and (4) peer-reviewed articles published in English before 1 February 2022. Studies were excluded if the retrieved item (1) was a review study, qualitative study, a single case report, an editorial comment, a meta-analysis of prior studies, or clinical trials under review; (2) animal study; (3) studies included children or patients aged above 75; (4) interventions for lower limb robotics or the use of lower limb exoskeletons and related robotic devices; (5) no investigation on the upper limb function as an outcome; (6) subjects with brain or neurological injuries other than cSCI; (7) consisted of abstracts with no associated full article published in a peer-reviewed English-speaking journal.

### 2.3. Study selection and data extraction

The titles and abstracts were imported into EndNote X9 to remove duplicated studies. After the removal of duplications, all records were manually screened by titles and abstracts to exclude irrelevant articles by two authors independently. Then, the same two authors independently performed a comprehensive extraction of key data points from those studies that met the eligibility criteria. All data were then extracted using a standard data collection form. Any discrepancies during the data extraction process were adjudicated by a third author. The following data were recorded using a table from each eligible article: (1) the name of the first author, (2) year of publication, (3) study design, (4) number of patients, (5) patient characteristics, (6) intervention, (7) type of robotic device, (8) outcome measured, (9) SCI stage and level, and (10) findings.

### 2.4. Quality assessment of individual studies

The selected studies were appraised by two independent reviewers (JSWH and GCWM) for methodological quality prior to inclusion in the overview, using a standardized critical appraisal tool, JBI Critical Appraisal Checklist for Systematic Reviews and Research Synthesis (33). Any disagreements that arise between the two reviewers will be resolved through consensus and discussion or guidance from a third reviewer (SWL) will be employed. A narrative summary of the results of the critical appraisal of systematic reviews will be presented and supported by relevant supporting tables. A score of 0–3 representing very low-quality; a score of 4–6 representing a low-quality; a score of 7–9 representing a moderate-quality; and a score of 10–11 will be considered as high-quality.

### 2.5. Data presentation and data analysis

Due to the methodological and clinical heterogeneity of patient groups, data pooling and meta-analysis were not performed. Various variables collected with absolute numbers and corresponding percentages were displayed for each study. A descriptive statistical analysis of the data collected was performed.

## 3. Results

**Figure 1:**
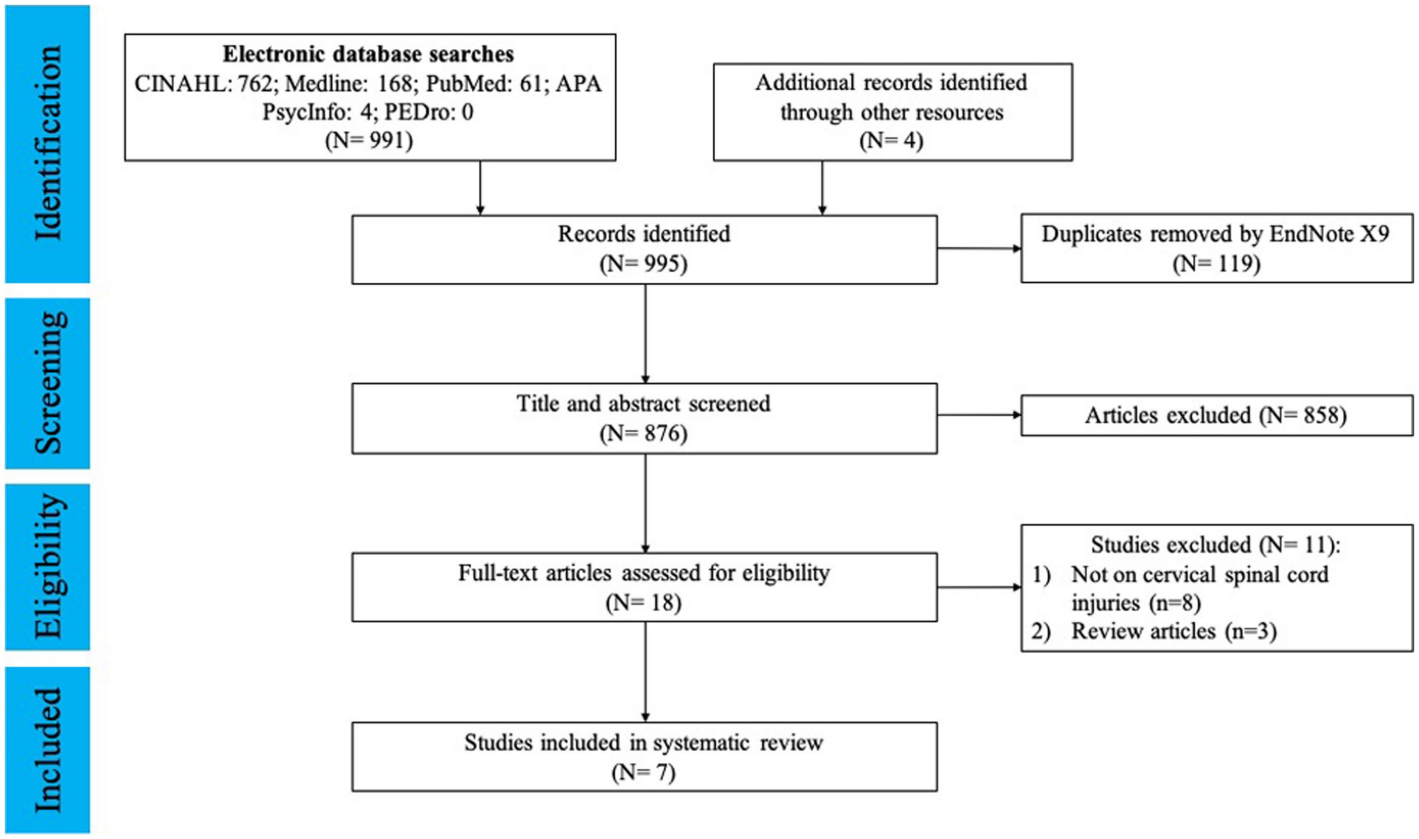
PRISM flowchart.

This systematic review includes items published from January 2010 to January 2022. After the removal of duplicates and articles that did not meet selection criteria, a total of 7 studies (of which four case series, one randomized controlled trial (RCT), and two quasi-experimental studies) were included, concerning a total of 87 subjects. The PRISMA 2020 flow diagram of the search process is shown in Figure 1.

### 3.1. Study selection and characteristics

In the preliminary search using the specified keywords on the databases, 995 articles were identified. After removing the duplicates and screening the titles and abstracts, 18 items (2%) were retained for full-text analysis by our two expert reviewers, of which 11 (61%) were excluded because they failed to meet inclusion/exclusion criteria. Of the 11 excluded items, they were removed due to various reasons (Figure 1). Among the 7 included studies, the sample size ranged from 4 to 34 participants. Characteristics of the included studies are summarized in Table 1.

**Table 1:** Characteristics and outcomes of studies included in the systematic review.

Article	Study Design	Aim	Sample Size (dropouts)	Participant characteristics	Intervention	Type of robotic device	Rehabilitation program	Outcome measures for robotic therapy	SCI stage and level	Findings
Zariffa et al. (34)	Multi-center Pilot Study	Establish feasibility of using robotic device in SCI inpatient setting and gather preliminary data on device’s efficacy	15 (3 dropouts)	14 M, 1 F	1 h × 3–5 days/wk. x 6 weeks along with combined conventional PT and OT exercises	Armeo Spring	Robotics with varying rehabilitation program	GRASSP	C4-6	Armeo spring increased the amount of rehabilitation training and reduced the time required from therapists but showed few functional benefits.
				19–75 years		Training mode: Passive assist		Action Research Arm Test		
								Grip Dynamometry	subacute	
								ROM		
Cortes et al. (35)	Case Series	To assess feasibility, safety, and effectiveness of robotic assisted training of upper limb in chronic SCI	10	8 M, 2 F	6 week wrist-robot training protocol (1 h/day × 3 times/wk)	InMotion 3.0 Wrist Robot	Robotics with varying rehabilitation program	Motor performance: Upper extremity motor score	C4-6	Robotic assisted training is feasible and safe that can enhance movement without affecting pain or spasticity in chronic SCI.
				17–70 years		Training mode: Passive, assistive, active, resistive		Pain Level (VAS)		
								Spasticity (Modified Ashworth Scale)		
									chronic	
Vanmulken et al. (36)	Case Series	To assess feasibility and effectiveness (arm-hand function and performance) of haptic robotic technology	5 (2 dropouts)	4 M, 1 F	1 h × 3 days/wk. x 6 wks	Haptic Master	Complex system with varying rehabilitation program	IMI and CEQ	C4-7	The haptic master is easy to work with and is feasible to use in patients with cSCI.
				25–70 years		Training mode: Passive assist, active assist			chronic	
Francisco et al. (37)	Case Series	To demonstrate feasibility, tolerability and effectiveness of RT in incomplete cSCI	10	8 M, 2F	Single degree of freedom upper limb exercises for 3 h per session, 3 times/wk. for 4 wks	MAHI Exo-II exoskeleton	Robotics with fixed rehabilitation program	Arm and Hand Function tests: Jebsen-Tylor Hand Function Test, Action Research Arm Test	C2-7	Repetitive training of arm movements with MAHI Exo-II is safe and has the potential to be used as rehabilitation intervention for patients with mild to moderate SCI upper limb impairments.
				19–60 years		Training mode: Passive assist, active assist		Upper Limb Strength: Upper limb motor score, grip, pinch strength		
								Independence in Daily Activities: Spinal Cord Independence Measure II		
									chronic	
Cappello et al. (38)	Case Series	To offer a fabric-based soft robotic glove as an assistive solution for participants with limited hand strength	9	8 M, 1 F	Administration of the Toronto Rehabilitation Institute Hand function test x2, once without glove for baseline line and once while wearing the glove	Fabric-based soft robotic glove	Robotics with fixed rehabilitation program	Upper Limb Function: TRI-HFT	C4-7	The fully portable robotic glove showed significant average object manipulation improvement and upper limb lift force.
				20–68 years		Training mode: Passive assist, active assist			Subacute/chronic	
Kim et al. (39)	RCT	To investigate the efficacy of upper extremity robotic rehabilitation as an adjunctive treatment to conventional OT in patients with tetraplegia.	34 (4)	28 M, 6 F	RT: OT with 30 min Armeo Power/day	Armeo Power	Complex system with varying rehabilitation program	Key muscles: Medical research council scale	C2-8	Small improvements in muscle strength and SCIM-III scores in RT group, but no statistically significant differences.
				RT: 56.7 + 13.6 years						
				OT: 47.1 + 14.9 years						
					OT: OT with additional 30 min OT/day	Training mode: Passive assist, active assist		Trained arm: UEMS	Subacute/chronic	
								SCIM-III		
Sorensen et al. (40)	Single-Subject Study (B-C-B)	To explore the impact of robotic training on upper limb function, ADL and training experience in subacute tetraplegic inpatients.	4	4 M	Six weeks total – two weeks of baseline OT followed by two weeks RT + PT then OT	Armeo Spring	Robotics with varying rehabilitation program	Arm and Hand Function: GRASSP	C4-7	Study could not confirm improvements were due to robotic intervention.
				19–62 years	11 sessions RT, 60 min each	Training mode: Passive assist		ADL: SCIM-III	subacute	
								Training Experience: 13-item Questionnaire		

ADL, activities of daily living; CEQ, credibility and expectancy questionnaire; cSCI, cervical spinal cord injury; EMG, electromyography; GRASSP, the graded redefined assessment of strength, sensation and prehension; IMI, intrinsic motivational inventory; OT, occupational therapy; PT, physiotherapy; RT, robotic therapy; ROM, range of motion; SCIM-III, spinal cord independence measure; TRI-HFT, Toronto Rehabilitation Institute Hand Function Test; UEMS, upper extremity motor score; VAS, visual analog scale.

### 3.2. Quality assessment

Studies were critically appraised using study-specific Joanna Briggs Institute (JBI) critical appraisal tools (CATs) to assess the methodological quality of studies in allied health literature. The 7 included studies were critically appraised by two independent reviewers using the JBI checklist. Of the 4 case studies (maximum quality score 10), 2 studies were assigned a score of 7 (35, 37), one study scored 6 (36), and another study scored 4 (38). The RCT studies scored 8 (maximum quality score 13) (39), while the two quasi-experimental studies (maximum quality score 9), the studies scored 6 (34), and 4 (40), respectively. Failure in blinding subjects and blinding therapists are the two most common methodological limitations in all included studies. The detailed results of the methodological quality assessment done with JBI assessment are shown in Tables 2–4.

**Table 2:** JBI critical appraisal checklist for case series.

Author	1	2	3	4	5	6	7	8	9	10	Total	Quality
Cappello et al. (38)	U	Y	U	N	Y	U	U	U	Y	Y	4/10	Low
Cortes et al. (35)	Y	Y	U	N	Y	Y	U	Y	Y	Y	7/10	Moderate
Francisco et al. (37)	Y	Y	Y	N	Y	Y	U	N	Y	Y	7/10	Moderate
Vanmulken et al. (36)	Y	Y	N	N	Y	Y	U	U	Y	Y	6/10	Low

Key: Y, yes; N, no; U, unclear. Questions: 1. Were there clear criteria for inclusion in the case series? 2. Was the condition measured in a standard, reliable way for all participants included in the case series? 3. Were valid methods used for identification of the condition for all participants included in the case series? 4. Did the case series have consecutive inclusion of participants? 5. Did the case series have complete inclusion of participants? 6. Was there clear reporting of the demographics of the participants in the study? 7. Was there clear reporting of clinical information of the participants? 8. Were the outcomes or follow up results of cases clearly reported? 9. Was there clear reporting of the presenting site(s)/clinic(s) demographic information? 10. Was statistical analysis appropriate?

**Table 3:** JBI critical appraisal checklist for quasi-experimental studies.

Author	1	2	3	4	5	6	7	8	9	Total	Quality
Sorensen et al. (40)	Y	U	Y	N	U	N	Y	Y	N	4/9	Low
Zariffa et al. (34)	Y	Y	Y	Y	Y	N	Y	U	N	6/9	Low

Key: Y, yes; N, no; U, unclear. Questions: 1. Is it clear in the study what is the ‘cause’ and what is the ‘effect’ (i.e., there is no confusion about which variable comes first)? 2. Were the participants included in any comparisons similar? 3. Were the participants included in any comparisons receiving similar treatment/care, other than the exposure or intervention of interest? 4. Was there a control group? 5. Were there multiple measurements of the outcome both pre and post the intervention/exposure? 6. Was follow-up complete and if not, were differences between groups in terms of their follow-up adequately described and analyzed? 7. Were the outcomes of participants included in any comparisons measured in the same way? 8. Were outcomes measured in a reliable way? 9. Was appropriate statistical analysis used?

**Table 4:** JBI critical appraisal checklist for randomised controlled trials studies.

Author	1	2	3	4	5	6	7	8	9	10	11	12	13	Total	Quality
Kim et al. (39)	Y	N	Y	N	N	Y	Y	U	Y	Y	U	Y	Y	8/13	Moderate

Key: Y, yes; N, no; U, unclear. Questions: 1. Was true randomization used for assignment of participants to treatment groups? 2. Was allocation to treatment groups concealed? 3. Were treatment groups similar at the baseline? 4. Were participants blind to treatment assignment? 5. Were outcomes assessors blind to treatment assignment? 6. Were outcomes assessors blind to treatment assignment? 7. Were treatment groups treated identically other than the intervention of interest? 8. Was follow-up complete and if not, were differences between groups in terms of their follow-up adequately described and analyzed? 9. Were participants analyzed in the groups to which they were randomized? 10. Were outcomes measured in the same way for treatment groups? 11. Were outcomes measured in a reliable way? 12. Was appropriate statistical analysis used? 13. Was the trial design appropriate, and any deviations from the standard RCT design (individual randomization, parallel groups) accounted for in the conduct and analysis of the trial?

### 3.3. Subjects: demographic and individual considerations

Taken together, all studies included a total of 87 subjects of both genders (72 men and 13 women), gender remained unclear or unreported for 2 subjects. Most patients were male (85%) and patients’ age ranged from 19 to 75 years old (mean average age at recruitment = 49 years old). Among these subjects recruited, 25 subjects were classified as having chronic SCIs, 19 subjects with sub-acute SCIs and 43 subjects with sub-acute to chronic SCIs. Whereas, the majority of the SCIs was found to be at C4–C5 (58.8%). These subjects were mainly recruited through rehabilitation centres’ databases, inpatient or outpatient units, research institutes’ referrals, or volunteering.

### 3.4. Intervention delivery

Robotic devices and training protocols varied across all studies. Interventions included the use of the following robotics: fabric-based robotic soft glove (*n* = 1), InMotion 3.0 wrist robot (*n* = 1), MAHI Exo-II exoskeleton (*n* = 1), Haptic Master (*n* = 1), Armeo Power (*n* = 1), and Armeo Spring (*n* = 2). Four robots are exoskeletons connected to multiple joint axes, which require modifications due to joint adaptations, and three robots are end effector devices connecting to distal parts of the joints, and are adaptable to those of various body sizes. In addition, three studies with both subacute and chronic patients in inpatient settings received co-therapy along with robotic therapy (34, 39), while other studies reported to receive only robotic therapy and did not specify if co-therapy was included. Robotic intervention was administered and supervised by physiotherapists (*n* = 2) or occupational therapists (*n* = 3), but 2 studies did not specify the background of their therapists and assessors (35, 38). The duration of the treatments ranged from 4 weeks to 6 weeks, and the length of training sessions between 30 min to 3 h. In addition, the characteristics and outcome parameters used in these studies were different (Table 1).

### 3.5. Outcome measures and statistics

Four studies focused on assessing the feasibility, safety, and effectiveness of UL RT in cSCI patients (34, 35, 37, 38). Another study focused on investigating the effectiveness of UL RT combined with conventional OT (39). The remaining two studies focused on the impact of UL RT in assisting upper limb functional activities for participants with decreased hand strength (36, 40).

Based on the evidence from two studies, it showed greater functional outcomes in participants that received RT in addition to conventional therapy (39, 40). One of these studies administered routine OT across both groups (39). An assessor blinded RCT was conducted to assess the effectiveness of robotic therapy to improve upper limb function in individuals with cSCI. The result showed intervention group showed small improvements in motor strength and SCIM-III scores in the RT group after 4 weeks of RT combined with conventional OT, but no statistically significant differences were identified between groups. Additionally, the study had an unequal distribution of participants between groups due to small sample sizes, where the number of acute patients were greater than chronic ones.

Two other studies (34, 40) showed slight improvements in upper limb function and independence with ADLs after RT, as well as patient satisfaction and enjoyment. However, they could not conclude that robotic rehabilitation brought functional benefits. The small number of participants and the lack of a control group limited the generalizability of findings.

Four case series (35–38) concluded that repetitive training of the affected arm using a robotic device was feasible and safe in enhancing upper limb movement in both subacute and chronic patient groups. While three of these studies found statistically significant improvements in motor performance, upper limb motor scores, grip and pinch strength, lift force, and ADLs after 6 weeks of robotic assisted training, a study from Vanmulken et al. (36) noticed diverse scores in intrinsic motivation, credibility, and expectancy among participants, potentially affecting their engagement with the robotic-assisted device. Additionally, some studies did not discuss their study limitations, and the long-term results of RT were unknown.

As all studies aimed to investigate the effectiveness of RT in improving upper limb function, between-group analyses were essential to compare the performance of both intervention and control groups pre- and post-treatment. All studies reported the patients’ baseline characteristics using descriptive statistics along with the mean and standard error (SE) or standard deviation (SD). Only 3 studies showed statistically significant data with *p*-values less than 0.05 (35, 37, 39). However, these studies failed to report CIs and only reported the *p*-values, increasing the likelihood of data misinterpretation and potential errors in accepting or rejecting the null hypothesis.

## 4. Discussion

This review was based on 7 studies that met the inclusion criteria. Among four out of these seven studies, participants with cSCIs demonstrated significant improvements in UL function, strength, grasping, and overall motor function with the implementation of RT as a primary intervention. Additionally, these studies found that repetitive UL arm training is feasible and safe for both subacute and chronic patient groups. Interestingly, patients with mild to moderate impairments showed better improvements in outcome measures when undergoing repetitive UL arm training than those with severe impairments.

Previous reviews mainly provide a broad overview on the clinical application, feasibility, and outcomes of RT alone (30, 31). They summarized that robotic assisted therapy (RAT) was shown to be feasible, safe, reduced therapists’ active assistance, and had positive effects on arm function and movement quality when compared to conventional therapy alone. However, they concluded that little to no clinically significant improvements in muscle strength, grip strength, ROM, and functional activity. Although our review also showed similar findings, we also focused on the importance of implementing RT into allied health rehabilitation.

The findings from this review suggested that Allied Health Professionals (AHPs) should implement RT alongside conventional therapy as part of their rehabilitation program. This can be implemented by instructing participants to perform UL functional exercises with the use of upper extremity robots providing resistance and movement assistance to the affected limb (9). Though there are difficulties in reaching a consensus regarding the appropriate dose, frequency and optimal robot for rehabilitative training, the effectiveness of RT may impact current guidelines that do not have recommendations for robotic rehabilitation in the management of cSCI.

To minimize active-therapist time required and resources, long-term follow-ups can be utilized in group sessions to increases efficiency in care delivery. Clinical research can also help to reinforce the importance of patient-centered interventions and determines the effectiveness of treatment given (41). Engaging with developments in research, the evidence provided can help to introduce new clinically and cost-effective ways to respond to patients’ needs. As PT practice aims to select and plan appropriate interventions to facilitate and restore movement and function and OT practice aims to help patients lead independent and productive lives, the findings of this review would can benefit current practice by providing AHPs with valuable insight into the effectiveness of RT as potential intervention for cSCI.

Concerning the type of intervention proposed, a very high variability was recorded in terms of robotic devices, the number of sessions per day, session duration, frequency, and joint involvement. Despite this, the lack of CIs in all studies would also increase the likelihood of statistical errors in data interpretation to decrease the credibility of the studies’ findings. Although Kim et al. (39) found small improvements in motor strength and functional independence in the RT group, the differences between the groups was not statistically significant. Similarly, Sorensen et al. (40) and Zariffa et al. (34) failed to demonstrate correlation between RT and UL function. Importantly, the lack of long-term follow-up in most studies can led to challenges in determining the continuous effects of RT or lasting changes of UL function. Hence, further study should focus on the long-term effects of RT as support toward clinical benefit. Likewise, by implementing long-term follow-ups by re-assessing participants through a variety of objective measures, it can allow AHPs to observe changes in UL strength and overall performance. As supported by Cortes et al. (35), it demonstrated RT allows functional gain to be retained over time. Additionally, understanding the participants’ and caregivers’ perceptions of RT using qualitative methods, such as focus groups and interviews, will help to supplement a clearer view of service users’ personal experiences.

Uncertainty and debating opinions around optimal robotic design limits the relevance and accessibility of robotic interventions for cSCI rehabilitation. Such factors include cost, patient satisfaction, user friendliness, comfort, convenience, time required for device set up, and its accuracy in providing repetitive UL training would need to be accounted. Implementing patient-centered designs by understanding the service users’ needs allows the multidisciplinary team to design new robots or modify existing ones to tailor the needs of AHPs and service users. Robotic devices that are cost-effective, quick to setup, and allow multi-joint training are highly preferred for cSCI rehabilitation design (17). Hence, future studies should adopt rigorous outcome measures to gain deeper insight into the cost-effectiveness and accessibility of RT amongst AHPs as an intervention for participants with cSCI. Additionally, educational opportunities including training courses and in-hospital teaching seminars, and multidisciplinary team discussions regarding the development of RT can be incorporated to further equip AHPs with the knowledge and resources needed to implement RT in cSCI rehabilitation.

Furthermore, there does not seem to be sufficient research tackling on the effectiveness of RT in improving UL function in specific population groups, especially in elderly patients with central cord syndrome, the most common incomplete cSCI. To address this, conducting clinical trials with larger sample sizes focusing on elderly patient groups is highly recommended to ensure more well-rounded evidence. On the other hand, Ross et al. (42) have identified potential barriers in RCT recruitment that may lead to difficulties in recruiting specific cSCI patient groups for conducting such setup. Such that, participants with strong preferences in receiving the intervention or conventional therapy may drop out from the study when knowing they might be placed into the “sham control group” when randomization is involved. Crossover studies randomize patients to a sequence of treatments may facilitate intra-individual comparisons. This study design often requires a smaller sample size for the same statistical power compared to parallel designs, and are thus less costly. However, crossover studies are only feasible when the condition being studied is relatively stable and the intervention has a short-term effect (43). While for most robotic rehabilitation for sSCI, the intervention might not be direct to show relative stability and might require a long duration to observe such effect. Additional expenses or inconveniences, such as travelling costs and transportation difficulties, may also lead to barriers that affect participants with disabilities. Further studies should tailored on patient recruitment in accordance with the participants’ needs, experiences and environment to minimize the number of dropouts and allow active patient participation in clinical trials (e.g., gender and ethnicity) (44). Moreover, researchers should also convey study information in a combination of oral, written and video methods along with professional advice from clinicians to ensure patients’ understanding toward the study procedures and associated risks (45).

## 5. Limitations

The strength of this review was the implementation of a thorough search strategy across five databases. In addition, the JBI manual provided a comprehensive guide to conducting this systematic review. However, there remains a number of limitations that should be mentioned when interpreting our results. Firstly, the current review only includes studies published in English. The results from relevant studies published in other languages were not accounted for and could affect the outcomes of our analyses and interpretations. Secondly, the small sample sizes and lack of control groups in selected case series and non-RCTs may lead to difficulties in assessing the methodological quality, risk of bias, and generalizability of results. Thirdly, as most of the included studies were retrospective and prospective in design, the limited RCT and experimental studies can limit the identification of cause-and-effect relationships between factors. Moreover, the studies included were mainly appraised as low-quality data which can inherently lead to review bias.

## 6. Conclusion

This scoping review provided an overview of evidence relevant to the effectiveness of AHP-prescribed RT in improving UL function for individuals with cSCI. Among the three seemingly average quality studies, it showed no significant effects on treatment outcomes. However, short-term results from selected studies demonstrated improvements in muscle strength and UL function. This may indicate the potential of AHPs to be incorporated as RT during cSCI rehabilitation. And from the findings in three medium quality studies, it appeared that robotic therapy coupled with strengthening exercises or conventional physiotherapy can yield greater significant improvement than RT alone. However, further study with control group and proper blinding protocol would still be needed. In addition, further research on the long-term effects of UL RT, its cost-effectiveness and accessibility, protocol development, and service users’ experience, would be essential to provide clinicians with a well-rounded perspective of both the clinical effectiveness and service users’ experience prior to incorporating UL RT as a standard clinical regime.

## Author contributions

Material preparation, data collection, and analysis were performed by JH, KK, SL, and GM. The first draft of the manuscript was written by JH and GM. SL and GM supervised the study. All authors contributed to the article and approved the submitted version.

## Funding

The authors would like to acknowledge the Health and Medical Research Fellowship Scheme 2019 (Grant number: 05190047) awarded to GM for supporting this study.

## Conflict of interest

The authors declare that the research was conducted in the absence of any commercial or financial relationships that could be construed as a potential conflict of interest.

## Publisher’s note

All claims expressed in this article are solely those of the authors and do not necessarily represent those of their affiliated organizations, or those of the publisher, the editors and the reviewers. Any product that may be evaluated in this article, or claim that may be made by its manufacturer, is not guaranteed or endorsed by the publisher.
