# Supplementary material for: The effectiveness of robotic-assisted upper limb rehabilitation to improve upper limb function in patients with cervical spinal cord injuries: a systematic literature review
Source: Front Neurol. 2023 Aug 9;14:1126755. doi: 10.3389/fneur.2023.1126755 (PMC10445651; doi:10.3389/fneur.2023.1126755)
Supplement: Supplementary file 1 [file Table_1.DOCX]

**Supplementary 1**

Search strategy in Pubmed (Jan 3^rd^, 2022)

Filter: from 2010

| Search | Query | Results |
| --- | --- | --- |
| #1 | **“Robotic therapy or Robotic assisted training or robotic assisted therapy or robot* or exoskeleton or telerobot* or wrist-robot* or robotic upper limb rehabilitation” AND “adult* or patient or individual* or young person* or young adult* or person or elderly or aged or older or elder* or geriatric* or elderly people or old people or older people or senior*” AND “cervical sci or cervical spine cord* or central cord syndrome or central spinal cord or central spinal cord injur* or ccs” AND upper limb or upper limb function or arm function or hand function or upper extremit* or upper extremity function** | 61 |
|  | Short-form abbreviations such as “SCI”, and wildcard symbol “*” were also used to expand the search. |  |

Search strategy in Medline (Jan 3^rd^, 2022)

Filter: from 2010

| Search | Query | Results |
| --- | --- | --- |
| #1 | **“Robotic therapy or Robotic assisted training or robotic assisted therapy or robot* or exoskeleton or telerobot* or wrist-robot* or robotic upper limb rehabilitation” AND “adult* or patient or individual* or young person* or young adult* or person or elderly or aged or older or elder* or geriatric* or elderly people or old people or older people or senior*” AND “cervical sci or cervical spine cord* or central cord syndrome or central spinal cord or central spinal cord injur* or ccs” AND upper limb or upper limb function or arm function or hand function or upper extremit* or upper extremity function** | 168 |
|  | Short-form abbreviations such as “SCI”, and wildcard symbol “*” were also used to expand the search. |  |

Search strategy in EMBASE (Jan 3^rd^, 2022)

Filter: from 2010

| Search | Query | Results |
| --- | --- | --- |
| #1 | **“Robotic therapy or Robotic assisted training or robotic assisted therapy or robot* or exoskeleton or telerobot* or wrist-robot* or robotic upper limb rehabilitation” AND “adult* or patient or individual* or young person* or young adult* or person or elderly or aged or older or elder* or geriatric* or elderly people or old people or older people or senior*” AND “cervical sci or cervical spine cord* or central cord syndrome or central spinal cord or central spinal cord injur* or ccs” AND upper limb or upper limb function or arm function or hand function or upper extremit* or upper extremity function** | 696 |
|  | Short-form abbreviations such as “SCI”, and wildcard symbol “*” were also used to expand the search. |  |

Search strategy in CINAHL (Jan 3^rd^, 2022)

Filter: from 2010

| Search | Query | Results |
| --- | --- | --- |
| #1 | **“Robotic therapy or Robotic assisted training or robotic assisted therapy or robot* or exoskeleton or telerobot* or wrist-robot* or robotic upper limb rehabilitation” AND “adult* or patient or individual* or young person* or young adult* or person or elderly or aged or older or elder* or geriatric* or elderly people or old people or older people or senior*” AND “cervical sci or cervical spine cord* or central cord syndrome or central spinal cord or central spinal cord injur* or ccs” AND upper limb or upper limb function or arm function or hand function or upper extremit* or upper extremity function** | 762 |
|  | Short-form abbreviations such as “SCI”, and wildcard symbol “*” were also used to expand the search. |  |

Search strategy in APA PsycInfo (Jan 3^rd^, 2022)

Filter: from 2010

| Search | Query | Results |
| --- | --- | --- |
| #1 | **“Robotic therapy or Robotic assisted training or robotic assisted therapy or robot* or exoskeleton or telerobot* or wrist-robot* or robotic upper limb rehabilitation” AND “adult* or patient or individual* or young person* or young adult* or person or elderly or aged or older or elder* or geriatric* or elderly people or old people or older people or senior*” AND “cervical sci or cervical spine cord* or central cord syndrome or central spinal cord or central spinal cord injur* or ccs” AND upper limb or upper limb function or arm function or hand function or upper extremit* or upper extremity function** | 4 |
|  | Short-form abbreviations such as “SCI”, and wildcard symbol “*” were also used to expand the search. |  |
